# 

**Published:** 1887-10-22

**Authors:** 


					f
No. 56; Vol. III.
October 22nd, 1887.
[Registered at the General Post Office
as a Sewiipaper.]
Per Annum, 6s. 6d.,post free.
Monthly Parts, 6d.; post free, 7Jd.
Ditto per Ann., 7s. 6d., post free.
JHcMntw, attit fljtlaittljnipg.

				

